# Iterative Situated Engagement Perspective: Meaning-Making Challenges Across Cancer Screening Phases

**DOI:** 10.3390/cancers17122007

**Published:** 2025-06-16

**Authors:** Daniela Lemmo, Maria Luisa Martino, Roberto Bianco, Anna Rosa Donizzetti, Maria Francesca Freda, Daniela Caso

**Affiliations:** 1 Department of Humanities, University of Naples Federico II, 80138 Naples, Italy; daniela.lemmo@unina.it (D.L.); marialuisa.martino@unina.it (M.L.M.); roberto.bianco@studenti.unina.it (R.B.); annarosa.donizzetti@unina.it (A.R.D.); fmfreda@unina.it (M.F.F.); 2 Department of Mental, Physical Health and Preventive Medicine, University of Campania Luigi Vanvitelli, 80138 Naples, Italy

**Keywords:** engagement phases, healthcare decision-making, cancer screening pathway, iterative situated engagement perspective, framework method, clinical psychology

## Abstract

**Background/Objectives:**Breast and cervical cancer screening programs are essential for early detection and timely treatment, yet participation rates remain suboptimal. Within a patient-centered care approach, engagement is increasingly viewed as a dynamic and emotionally grounded process. The literature conceptualizes three phases of engagement in healthcare decision-making: ‘recruit’, ‘retain’, and ‘sustain’. When these phases intersect with the structured pathway of cancer screening, they generate specific meaning-making challenges that shape how women relate to prevention and care. This study adopts the lens of Iterative Situated Engagement (ISE) to explore how women experience and negotiate these challenges, differentiating them across the three engagement phases. **Methods**: A theory-driven qualitative design was adopted. Data were collected through semi-structured interviews with 40 women aged 25–69 years participating in public breast and cervical cancer screening programs. Thematic analysis was conducted using the Framework Method. **Results**: In the ‘recruit’ phase, engagement was driven by Cancer Risk Monitoring, Self-care Motivation, Fear of Death Management, and Coincidence. The ‘retain’ phase emphasized Trust in Healthcare Providers, Accessibility of Services, Recurrent Invitations, and Informal Result Previews. About the ‘sustain’ phase, Continuity of Healthcare Providers, Driving Best Practices Dissemination, Flexible Organization of Healthcare Services, and Shorter Waiting Times for Results were highlighted as key factors in maintaining engagement over time. **Conclusions**: Women’s engagement in cancer screening emerges as a dynamic, multi-phase process shaped by psychological, emotional, and organizational levels. These findings contribute to the development of the ISE conceptual proposal, which frames participation as an iterative, situated, and meaning-making trajectory. Strengthening personalized health communication and improving the coordination of primary care services could enhance sustained participation in screening programs, supporting strategies to reduce health disparities and promote preventive practices**.**

## Simple Summary

Cancer screening programs help detect breast and cervical cancers early, improving the chances of successful treatment. However, many women do not consistently participate in these programs. This study explores the meaning-making challenges women encounter across the engagement phases of the cancer screening pathway. These challenges are shaped by emotional, psychological, and organizational factors, which influence how women construct meaning and navigate the phases of ‘recruit’, ‘retain’, and ‘sustain’. Through interviews with women aged 25 to 69, we examined how they construct different meanings across the engagement phases of ‘recruit’, ‘retain’, and ‘sustain’. Drawing on a conceptual proposal—Iterative Situated Engagement (ISE)—we understand engagement not as a single decision, but as an iterative, situated, and narrative process. Understanding how women experience and construct meaning around their engagement can help improve communication strategies, healthcare organization, and primary care practices, ultimately supporting greater participation in preventive health programs.

## 1. Introduction

Breast and cervical cancer screening programs are essential public health interventions designed to detect early-stage disease or pre-cancerous conditions in asymptomatic individuals, enabling timely diagnosis and treatment [1]. Breast cancer remains the leading cause of cancer diagnoses among women, followed by cervical cancer. Recent estimates indicate that approximately 30% of new cancer diagnoses derive from screening activities, significantly improving the five-year survival rates—90% for breast cancer and 79% for cervical cancer [1,2,3,4].

According to the Italian National Screening Observatory [5], cancer screening programs are included in the Essential Levels of Assistance (“Livelli Essenziali di Assistenza”-LEA), aimed at guaranteeing citizens’ right to health protection. National healthcare systems have committed to promoting equity in access to preventive services by systematically inviting the target population to participate in organized screening programs [6]. Specifically, women aged 50–69 years are invited every two years to undergo a bilateral mammogram, while women aged 25–64 years are invited every three years for a PAP smear test, both offered free of charge. Nevertheless, participation rates remain suboptimal: only 41% of eligible women attend mammography screening, and only 28% participate in cervical screening programs, with even lower rates observed in Southern Italy [6].

These data highlight a gap between the proven medical efficacy of screening programs and individual healthcare decisions regarding participation. In understanding decision-making processes related to cancer screening, the literature identifies numerous psychological determinants operating at the individual, emotional, and relational levels, which can either promote or hinder participation [7,8,9,10].

Within this context, the framework of Shared Decision-Making (SDM) has become increasingly relevant. SDM is considered a cornerstone of patient-centered care [11], offering a structured process that captures the complexity of healthcare decisions, particularly those classified as preference-sensitive—decisions where benefits and harms are closely balanced and the choice depends on individual values and preferences [12,13]. Through SDM, a bidirectional flow of information between healthcare providers and patients enables more informed and personalized choices [14,15,16].

Specifically, SDM is built upon three fundamental components: (a) access to information about the disease, as well as the available screening and treatment options; (b) consideration of individual values, beliefs, and attitudes towards health and prevention; and (c) active patient involvement in the decision-making process alongside healthcare professionals [17].

Numerous factors influence decision-making about cancer screening participation, including pre-existing knowledge about the examinations, awareness of cancer risk factors, emphasis on the benefits of an early diagnosis, and emotional responses such as fear of having cancer [18,19,20]. Additionally, the role of healthcare actors is crucial: individuals often rely on trusted figures—physicians, healthcare professionals, or even pharmacists—to support their screening decisions [21,22]. Clear, consistent recommendations and the availability of a dedicated space to address concerns are important facilitators of screening participation [23,24,25,26,27,28,29]. On the other hand, embarrassment, shame, fear of pain, and fear of a positive result emerge as major emotional barriers, particularly regarding mammography and PAP smears [30,31,32,33,34,35].

The original definition of SDM by Charles et al. (2006) [36] emphasizes the need to distinguish between shared decision-making and informed decision-making. Within the SDM framework, engagement is recognized as a fundamental aspect: moving beyond the traditional asymmetry in healthcare interactions, SDM encourages active patient participation and responsiveness to individual expectations and needs [37,38].

In the context of cancer screening, where healthcare decisions are highly sensitive to individual preferences, understanding how patients engage with the process—and how the healthcare system can support this engagement—is critical to enhancing participation rates and promoting effective personalized prevention pathways.

**1.1.** **Rethinking** **Engagement Phases in the Cancer Screening Pathway: A Process Within the Process**

The construct of engagement has traditionally been framed within the Shared Decision-Making (SDM) model; however, when considering preventive practices, it should not be confined to the clinical encounter between patient and professional. Rather, engagement in prevention should be understood as an ongoing relational process between healthcare systems and individuals, aimed at fostering the adoption of active health behaviors [39,40]. This perspective shifts the paradigm from “What is the matter?” to “What matters to you?” [41], placing individual agency at the core of health promotion, prevention, and illness management [42]. In line with this vision, the SDM model has been increasingly applied to preventive settings as a strategy to support personalized dialog and shared responsibility in health-related decision-making [43,44].

A key contribution in the literature is the Patient Health Engagement (PHE) model, developed by Graffigna and colleagues [45]. The PHE model offers a systemic and psychological view of engagement as a dynamic process that unfolds through progressive phases of activation, each characterized by specific configurations of thoughts, emotions, and actions. One of the defining features of this model is its emphasis on the emotional and identity-related dimensions of the patient experience. It recognizes emotional processing and the reconstruction of a health-related self-image as central components of engagement. This approach makes it possible to go beyond a linear perspective of involvement, which focuses solely on behavioral compliance or cognitive decision-making [46].

Building on these theoretical foundations, we propose a new conceptualization—Iterative Situated Engagement (ISE)—which maintains continuity with the processual and experiential vision of the PHE model, while adding an epistemological framework of narrative meaning-making [47,48,49,50]. ISE defines engagement as a “process within a process”: an iterative, situated, and narrative process that reactivates at each phase of the care pathway, giving rise to specific meaning-making challenges that individuals must navigate in order to maintain their sense of agency and coherence. From this standpoint, engagement is not merely the result of progressive cognitive, emotional, and behavioral activation, but rather emerges from the ongoing negotiation of meaning that individuals undertake within specific clinical and relational contexts.

This perspective is particularly salient in the context of cancer screening programs, which unfold through a series of distinct procedural phases—each of which poses unique narrative and emotional challenges that demand new acts of meaning-making. Screening is not a single clinical event, but rather a complex, multifactorial pathway encompassing invitation, organization, execution, result interpretation, diagnostic follow-up, and ongoing surveillance [51]. Each of these phases constitutes a psychological threshold [52], where individuals renegotiate their relationship with prevention—especially in light of the emotional vulnerability elicited by diagnostic procedures [53,54,55]. Furthermore, the recent literature underscores the need to view women’s engagement in screening as part of a broader healthcare trajectory rather than an isolated occurrence. Experiences such as obstetric violence during childbirth or psychological distress related to cancer diagnosis and treatment can deeply affect trust in healthcare systems and, consequently, shape future participation in preventive care [56]. Exploring engagement across different stages of care may therefore offer deeper insights into the psychological and relational factors shaping women’s preventive behaviors.

To better understand how individuals engage throughout this complex process, we draw on the model proposed by McCarron et al. [57], which delineates three core phases of engagement within healthcare pathways:

• Recruit: the initial phase, in which individuals decide to participate (“Why did I decide to participate?”);
• Retain: the phase concerning the maintenance of adherence (“Why do I continue to participate?”);
• Sustain: the phase that addresses the support required to ensure long-term involvement (“What do I need to keep participating?”).

These phases do not follow a strict linear progression; rather, they represent dynamic psychological configurations that may emerge, recur, or shift at various points along the care pathway—each accompanied by specific meaning-making challenges that reflect shifts in motivation, vulnerability, and trust.

Engaging in screening programs is therefore not merely a set of clinical tasks, but a lived narrative through which individuals continuously renegotiate their agency and meanings in relation to themselves, to health, and to the healthcare context. Engagement unfolds across the phases of the cancer screening journey—‘recruit’, ‘retain’, and ‘sustain’—and is continuously reshaped through lived experience, emotional regulation, relational dynamics, and organizational conditions. Each phase becomes a site of meaning negotiation, where women revisit, reinterpret, and reconfigure their motivations, vulnerabilities, and trust in the healthcare system—thus confronting meaning-making challenges that reshape their engagement journey.

**Figure 1:**
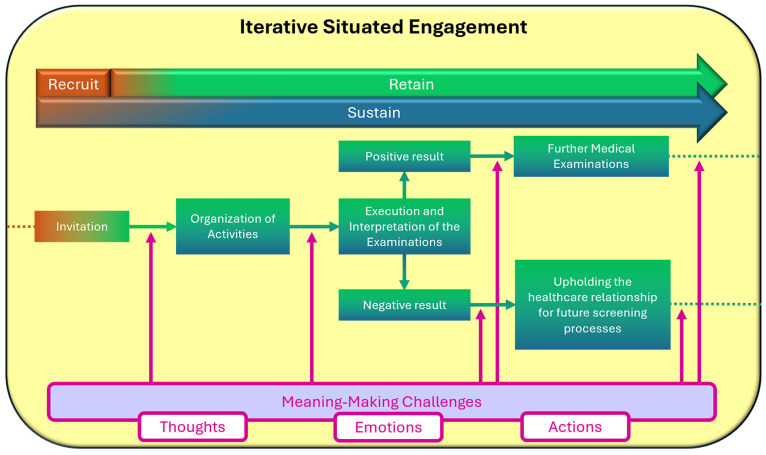
Iterative Situated Engagement Perspective in cancer screening pathway.

Within the Iterative Situated Engagement (ISE) framework, the term iterative captures the cyclical and adaptive nature of engagement: each experience does not merely follow from the previous one, but transforms it—opening new perspectives and eliciting novel emotional responses (Figure 1). Engagement is thus conceived as a temporal process of continuous re-signification, where pre-, during-, and post-screening experiences interweave and influence one another.

This perspective emphasizes that an individual’s position within the screening pathway is not static, but in constant flux—shaped by cognitive, affective, and institutional factors. Engagement in healthcare is not the result of a fixed identity or singular decision, but rather emerges through a series of iterative interactions, each conditioned by context and renewed at every point of contact.

The ISE model aligns with and complements existing frameworks—such as SDM, PHE, and patient activation—while offering a socio-constructivist and clinically grounded perspective focused on meaning-making, emotional elaboration, and relational co-construction. Moreover, its principles extend beyond screening, proving applicable to other areas such as chronic illness self-care, preventive health, and digital health interventions.

While the previous literature has highlighted the perspectives of healthcare professionals on the adaptation process between citizens and screening services [58], few studies have investigated the lived meanings of engagement from the citizens’ point of view across the distinct phases of the screening pathway [59,60].

In response to this gap, adopting a clinical psychological and socio-constructivist perspective, the present study aims to explore how women participating in public breast and cervical cancer screening programs construct meaning around their engagement experiences—specifically across the phases of ‘recruit’, ‘retain’, and ‘sustain’. Gaining insight into these meaning-making challenges can inform the development of more personalized preventive strategies, enhance participation rates, and support a truly person-centered approach to oncological screening programs.

## 2. Materials and Methods

### 2.1. Participants and Data Collection

This is a qualitative, theory-driven study adopting a thematic analytic approach based on the Framework Method [61], conducted in accordance with the COREQ (Consolidated Criteria for Reporting Qualitative Research) guidelines.

The study was carried out as part of the MIRIADE Project and involved Italian women residing in the Campania Region, who had recently participated in breast or cervical cancer screening programs provided by regional public healthcare services.

Participation in the study was offered by trained nurses or administrative staff during check-in procedures, immediately prior to the screening examination, following a standardized protocol implemented across all participating centers.

Eligibility criteria included:

• Being a woman aged 25–69 years (according to the Italian screening program guidelines) who underwent breast or cervical screening examinations in public facilities.

Exclusion criteria included:

• Having an age outside the eligibility range established by the regional screening programs.

Recruitment continued until data saturation was achieved, in line with qualitative research standards.

Interviews were conducted between January and July 2022, in a private setting provided by the local cancer screening services involved in the MIRIADE project. Each interview was carried out by qualified clinical psychologists trained in qualitative interviewing techniques, immediately after the women underwent their screening examination but before receiving the test results. Interviews lasted approximately 20–25 min, were audio-recorded with participant consent, and transcribed verbatim.

Participation was voluntary. All participants signed informed consent forms, including specific authorization for data processing in accordance with the GDPR EU 2016/679 and D.L. 101/2018. The study was conducted according to the guidelines of the Declaration of Helsinki and approved by the Ethics Committee of Psychological Research of the Department of Humanities of Federico II University, in Naples, Italy (prot. no 16/2022).

To explore the meanings women attribute to their engagement process in preventive practices, an ad hoc semi-structured interview guide was developed. The guide covered key areas drawn from the existing literature on health engagement, decision-making, and preventive behaviors. The interview aimed to explore the experience diachronically, across different phases of the screening process, and was administered flexibly to follow the women’s narrative flow and meaning-making processes.

The interview explored the following areas through open-ended prompts:

• Representation of health and risk (e.g., “What does health mean to you? How do you take care of your health?”)
• History of access to healthcare services (e.g., “How did you learn about the screening program? What motivated you to participate?”)
• Emotional experiences related to the examination process (e.g., “What sensations or emotions did you feel during the examination?”)
• Factors supporting ongoing engagement in screening (e.g., “What could help you continue participating in prevention programs?”)

A preliminary pilot test was conducted on a small subsample of participants to refine and validate the interview prompts.

The interviews were treated as a single, holistic narrative corpus. Two independent researchers (D.L. and M.L.M.) analyzed the data based on three key engagement questions derived from McCarron et al. [57].

### 2.2. Data Analysis

The data were analyzed following the Framework Method [61], a systematic approach for thematic analysis of qualitative data widely used in health research [62]. After transcription and familiarization with the material, two independent researchers (D.L. and M.L.M.) coded the transcripts line-by-line, applying descriptive labels to relevant text segments.

Following the initial coding, the researchers jointly developed an analytical framework by grouping codes into overarching thematic categories. These categories captured key concepts and recurring patterns of narrative challenges emerging from participants’ narratives, organizing the data not by frequency but by shared conceptual significance.

The thematic analysis was structured around three engagement phases derived from McCarron et al. [57]: ‘recruit’ (“Why did I get engaged?”); ‘retain’ (“Why do I stay engaged?”), and ‘sustain’ (“What do I need to continue being engaged?”).

This theory-driven organization provided a guiding framework for the analysis while allowing thematic categories to emerge inductively from participants’ lived experiences and meaning-making challenges within each engagement phase.

Although a thematic analysis was conducted using the Framework Method; the categories were developed not only in order to organize recurring content, but also to capture the meaning-making challenges expressed in women’s narratives about their engagement in cancer screening practices. While systematic and structured, this method also recognizes the centrality of interpretation in the qualitative analysis phase. Specifically, the themes emerging from the analysis are understood as “interpretative concepts or propositions that describe or explain aspects of the data”, developed through iterative comparison within and across cases. In our study, we applied the Framework Method in a way that balanced a deductive approach—grounded in the aforementioned theoretical models—with an inductive process that allowed novel challenges to emerge organically from the collected data.

Data were thus organized into inductively derived categories that reflected participants’ unique meaning-making within the three engagement phases. Inter-coder agreement was calculated by dividing the number of coding agreements by the total number of agreements and disagreements, reaching a 90% agreement rate across the entire corpus.

Although formal member-checking was not conducted due to the timing and structure of data collection, credibility was ensured through triangulation strategies. These included collaborative discussions among researchers from different disciplinary backgrounds, iterative comparisons of coding, and peer debriefing sessions to critically review interpretations and enhance the overall trustworthiness of the analysis.

All procedures adhered to the COREQ guidelines for qualitative research, ensuring rigor, transparency, and trustworthiness throughout the study process.

## 3. Results

A total of 40 women participated in the study, equally divided between cervical and breast cancer screening groups. Participants were recruited through public healthcare services. The mean age of the total sample was 56.0 years (SD = 11.4; range: 25–69). The cervical screening group had a mean age of 49.0 years (SD = 7.5; range: 25–64), and the breast screening group had a mean age of 63.0 years (SD = 5.2; range: 50–69). Detailed demographic and contextual characteristics are reported in Table 1.

**Table 1:** Demographic and contextual characteristics of the participants.

Group	Mean Age (Years) ± Standard Deviation (SD)	Age Range (Years)	Screening Context
Total Sample (N = 40)	56.0 ± 11.4	25–69	Public Healthcare
Cervical Screening Group (N = 20)	49.0 ± 7.5	25–64	Public Healthcare
Breast Screening Group (N = 20)	63.0 ± 5.2	50–69	Public Healthcare

The thematic analysis, structured around the engagement phases of ‘recruit’, ‘retain’, and ‘sustain’ [57] identified distinct meaning-making categories characterizing each stage. These categories reflect the key psychological and emotional factors through which women construct and negotiate their engagement with cancer screening practices over time. The results, organized by each phase of the engagement process, are reported below.

### 3.1. Meaning-Meaking Challenges of ‘Recruit’ Phase Engagement Cancer Screening

Regarding the ‘recruit’ phase, the results show that in 70% of cases, the engagement begins by invitation (phone call or letter) from the NHS. In 15% of cases, it is because of word of mouth between friends or acquaintances. In 15% of cases it is because of spontaneous engagement, after having independently learned of the initiative through online research or advertising. The women described different ways of making meanings of their initial engagement with cancer screening programs.

Four meaning-making challenges were identified:

• **Cancer Risk Monitoring:** This category organizes systems of meaning centered on the awareness of the possibility of falling ill on the hope of excluding the presence of the disease and, where inevitable, on the need to identify an oncological risk in good time, relying on medical expertise to coordinate any treatment process. This category concerns women who express a relationship with prevention understood as an action aimed at facing cancer risk (*“it takes structure and also strength to face the risks”*). In this narrative category we find experiences of tumors (not limited to breast or cervix tumors)—as well as other diseases (stroke, heart attacks) both in one’s own history and in the family history (*“for all that I have already experienced, discovering this risk in time is only an advantage”*). Therefore, risk becomes representable, tangible, and integrated into one’s own life. In this category, decisive for engagement is the awareness of vulnerability and the feeling of uncertainty regarding health, which can be reduced through preventive action. The body is represented as a machine that can fail, which therefore presents signs and symptoms that screening can help to identify and monitor. Preventive action is also guided by a relational motivation: the need to maintain a status quo of good health in continuity with the role of caring for others, especially children and the family. A responsibility emerges that intertwines the self with one’s relationships, signifying illness as pain and burden for family members (*“getting sick creates pain, it creates suffering, it also creates discomfort for others around you; therefore, prevention, first of all for yourself, but then also for others, that is, having respect for others too […] one who gets sick is- is just doing something wrong, but prevention helps everyone in the family feel good”*). Within this category, engagement is linked to the meaning of reducing uncertainty and controlling risks that are sometimes difficult to represent and thus generate anxiety.
• **Self-care Motivation:** This category organizes systems of meaning that consider self-care as an integrated process in one’s life, within which the relationship with cancer prevention is configured as a habitual practice for managing the maintenance of one’s health in a non-ambivalent way, using all the health opportunities that favor its implementation. In this category, decisive for engagement is the feeling of being involved in a preventive practice that is consistent with one’s health goals, in order to perceive a sense of control over one’s life (“*Health comes first. It is true that people neglect themselves, but prevention is fundamental. Precisely, it consists in the protection of the human being. In other words, we are not immortal”*). Health, from this perspective, represents the purpose of life to be preserved by assuming healthy lifestyles that align with a family habit of self-care (*“I am surrounded by doctors, by my husband, by my daughter who is about to graduate… there is the other who is a pharmacist. Let’s say I am surrounded by a healthcare environment, so we chew prevention every day”*). The responsibility for taking care of one’s own health is never delegated to others, and consistency is maintained in routine health checks (*“health should always come first, and it is important to check myself as much as I can. I do enough, that is, [I do] analyses every year, in short”*). Taking care of yourself and your health is configured as an action of self-love, loving yourself and giving value to your life. This aspect is intertwined with the need to have welcoming and available healthcare contexts, in which the healthcare relationship takes the form of an attentive relationship of taking charge and care. Risk is understood as something that depends on oneself, in the form of a personal action, a bad habit, or a vice to be avoided because it can lead to a threat to one’s health (*“there is one thing I do and I shouldn’t do, *i.e., *smoke, unfortunately, smoking… I do realize that this thing you do is unfortunately very wrong for health”*).

This category is more frequent in gynecological prevention, suggesting that cervical screening is seen as an appointment with one’s sexual health, as well as with an intimate and often little-known part of oneself (*“Even dedicating time for yourself, to do something for you, that goes outside of everyday life, because by doing it, even at fairly long intervals, you begin to establish relationships with that part of you and of your life that needs care and very often goes underestimated’*).

• **Fear of Death Management:**This category organizes systems of meaning related to an overwhelming fear of death, which finds a form of control in screening. This category of meaning frames cancer prevention as an attempt to regulate the anguish associated with images and thoughts related to the risk of death (*“Oh well, I always focus on that, always on death, that in any case I then leave the family alone… yes, in fact, I’m really terrified”*). This positioning also references the health emergency from COVID-19, which, from an institutional point of view, forced the cessation of screening visits, and from an individual point of view, generated greater health concerns (*“No, oh well, I’m a guy who, let’s say that after Covid I’ve become a bit hypochondriac, I’m afraid, I wasn’t like that, so I think it’s important to take care of myself and prevent, above all. I wasn’t like that before; I find myself a bit changed”*). In this category, decisive for engagement is the fear of a deadly disease such as cancer, which they try to keep under control by undergoing screening exams.
• **Coincidence:** This category organizes systems of meaning that are rather non-specific, in which a clear preventive intent does not seem to emerge. Together with a lack of knowledge of the program, in this category we find a passive-dependent dimension in the relationship. This category of meaning reflects sporadic and occasional engagement with cancer prevention, where the responsibility for healthcare is outsourced to medical professionals or family members (*“If I wasn’t called, I wouldn’t have come. I’m being honest, unfortunately”*). Such a form of recruitment seems to happen by virtue of the prompting of a significant other (institution or family) who urges one towards prevention, with an external prompting against the block caused by fear (*“I have never tried it because to tell the truth I am not [enough] spurred on this…always for the fear that something will come out. I have never been spurred on, while having the phone call, it is as if they had spurred you to make a gesture”*) or against the perceived irrelevance of the topic of prevention (*“if I wasn’t called, I wouldn’t have come. There’s no reason, I always postpone, that’s how I am […] If I have to think about this exam by myself, I really don’t think about it, I’ll pass on”*). Risk is represented as something over which one does not have much power, possibly because of direct or indirect medical past experiences of a negative nature.

### 3.2. Meaning-Meaking Challenges of ‘Retain’ Phase Engagement Cancer Screening

With respect to the ‘retain’ phase—and therefore to the maintenance of engagement in mammography and cervical screening practices—the results show that in 93% of the interviews, the intention to maintain one’s involvement over time is expressed. In 7% of the interviews, it has not been possible to trace the content.

Four meaning-making challenges were identified:

• **Trust in Healthcare Providers:**This category organizes systems of meaning that emphasize the perceived need to establish a trusting and respectful relationship with the healthcare provider, as experienced within the service, in order to ‘sustain’ engagement in preventive practices. Concerns about potentially unpleasant healthcare encounters—particularly among first-time attendees—are alleviated by professionals’ displays of kindness, respect, and confidentiality. These relational qualities foster trust in the institution and support a sense of being recognized as an individual with legitimate needs and concerns. A key relational factor in maintaining engagement in gynecological prevention is the presence of a female midwife or gynecologist, given the feelings of shame and tension often associated with the examination (*“I was afraid of meeting a cold or unwelcoming figure; instead the midwife was kind and immediately put me at ease….you know, the position of the exam doesn’t allow you to relax”*).
• **Accessibility of Services:**This category organizes systems of meaning related to all the access points the NHS provides in order to guarantee access to the service. Appointments are offered in a short time frame, so they can be more easily included in the daily activities. The chance to undergo a screening exam when users go to the service for other types of exams is also offered (*I came to take my husband for an ultrasound, I asked if it had been 3 years [since my last screening], and they immediately offered me to do a pap test*).
• **Recurrent Invitations:**This category organizes systems of meaning that highlight the need for continuity over time in relation to a well-functioning organizational healthcare model, in order to sustain engagement in preventive practices. The invitation by letter and even more by phone call is interpreted as “being in the mind of the other”: an institution that takes care of one’s health and urges you to remember the cyclical nature of screenings; it is also interpreted as an element perceived as personalization and support (*“In short, the phone call already makes you feel good. Because, in short, being called at home by the health institution… in fact I was a bit perplexed because the first time I came here, I came because of friends who told me, but this time with that phone call I was pushed and enticed more. They are so nice, they send me to be screened, both pap-tests and other things”*);
• **Informal Result Previews:**This category organizes systems of meaning that express the importance of the relational strategy of anticipations and reassurances that all operators provide during exams in order to make the anxiety for the results more manageable (*“They have been very kind and it is also satisfying, because they give explanations and this is already very useful, otherwise you wait for the report, and one is anxious with the agitation of waiting for this report… but [being able to] know everything beforehand is good, there are no problems and one already feels relieved”*).

### 3.3. Meaning-Making Challenges of ‘Sustain’ Phase Engagement Cancer Screening

Regarding the ‘sustain’ phase, is it intended as the set of indications provided as necessary in order to be able to support engagement in preventive practices in the future. The results reveal four narrative challenges that organize meanings related to the changes that could occur to improve the prevention experience and support sustained engagement in preventive practices.

Four meaning-making challenges were identified:

• **Continuity of Healthcare Providers:**This category organizes systems of meaning that expresses hypotheses of change relating to the medical personnel involved and dedicated to the screening process. The medical personnel involved should, if possible, always be the same over the years and along the various stages of the process. This aspect concerns the reliability of always being able to find the same operator even after years, as an element of guarantee and trust (*“often they are trainees and are constantly changing, and it is bad not always to find the same operator who knows you”*).
• **Driving Best Practices Dissemination:**This category organizes systems of meaning that express the importance of feeling themselves involved as ambassadors of good practices within informal networks of friendships and acquaintances. In this way, the category organizes meanings that refer to the possibility of remaining engaged in preventive practices. To sustain investment in prevention over time, the need and intention to deepen knowledge on the topic and to receive adequate health education emerges. This appears to be aimed at enabling the involvement of other women, particularly those who are hard to reach or who struggle to place trust in the healthcare system (*“I’m younger and I found it very useful, it’s important that I get the message across to those who don’t know you can come or don’t trust [the NHS]”*).
• **Flexible Organization of Healthcare Services:**This category organizes systems of meaning that express hypotheses of necessary change related to enlargements of the setting in terms of times and health practices offered during the screening. On the one hand, the importance of the extemporaneousness of the exam emerges, as an “occasion” that suddenly presents itself. On the other hand, there is the contingency of the screening offer compared to other health actions and therefore the possibility of carrying out more health actions together. Both hypotheses of change emerge as necessary to sustain engagement (*“It would be the case that, in addition to the pap test, an ultrasound can also be done [in conjunction with the PAP test], but also a complete gynecological visit”*).
• **Shorter Waiting Times for Results:** This category organizes systems of meaning related to results’ waiting time, and the time between one exam and another as foreseen by the program (2 years in the case of mammography screenings and 3 years in the case of pap smear screenings). The waiting time is not easily managed, and often expresses a preference for private screenings, as it allows for faster results. This seems to underline the necessity to shorten the waiting time (*“If after two weeks they don’t call me for the results, I’ll call. Just as I go to have a mammogram every year”*). Finally, this category expressed the need to undergo other medical tests (e.g., an ultrasound), thereby extending the meaning of the screening itself.

## 4. Discussion

**Figure 2:**
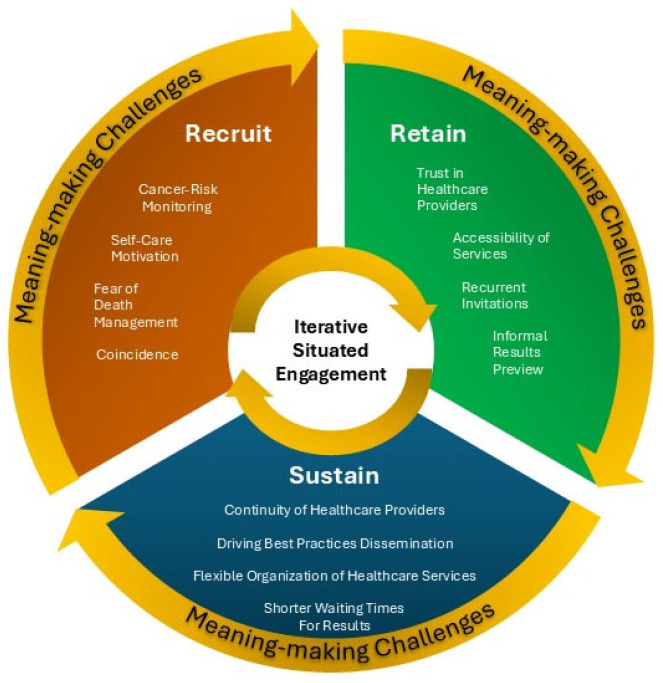
Meaning-making challenges for ‘recruit’, ‘retain’, and ‘sustain’ phases of engagement in breast and cervical cancer screening pathway.

As as illustrated in Figure 2, the results that emerged allow us to shed light on the meaning-making challenges underpinning women’s engagement in breast and cervical cancer screening practices at the local health authorities of the Campania Region, focusing on three engagement phases—’recruit’, ‘retain’, and ‘sustain’—as proposed by McCarron et al. [57].

Regarding the first phase, the ‘recruit’ phase—which, according to the ISE conceptual proposal, can be described as an initial narrative threshold corresponding to the moment of invitation to screening—our results identified four distinct meaning-making challenges: Cancer Risk Monitoring, Self-care Motivation, Fear of Death Management, and Coincidence. These narrative challenges characterize different subjective positions of women, which intersubjectively define the determinants of the beginning of the engagement process, thus establishing the field of preventive practices [63]. Such differentiation aligns with the literature recognizing the diversity of motivational and emotional factors influencing screening participation [10,18]. Notably, the Self-care Motivation category was particularly prominent among cervical screening participants, suggesting a broader conceptualization of preventive health tied to sexual and reproductive well-being. The Fear of Death Management category, contrary to some prior findings suggesting that fear inhibits preventive behaviors [9], highlighted how recognized and narrativized fear can act as a motivator for proactive engagement when emotionally elaborated. Our findings highlight that fear—particularly the fear of illness or death—can function as a double-edged psychological driver in preventive health practices. While in some instances it reflects deeper anxieties that hinder individuals’ capacity for rational engagement with healthcare services, in our study, fear also emerged as a motivational lever that encourages participation in screening. However, when fear becomes maladaptive, it may lead to emotional withdrawal, avoidance behaviors, or heightened psychological distress, ultimately compromising the effectiveness of preventive interventions. This distinction between paralyzing death anxiety and manageable death-related concerns offers new insights into emotional regulation mechanisms within preventive health behavior.

This phase marks the entry point into the screening journey and entails the initial construction of meaning around prevention and health risk, often reconnecting with past experiences. This highlights the importance of supportive communication and relational strategies within screening settings—strategies that recognize and address emotional vulnerability as integral to a person-centered approach to health promotion.

Regarding the second phase of engagement—the ‘retain’ phase—which, within the ISE conceptual proposal, can be understood as a stage of narrative consolidation in which women revisit and re-signify their initial engagement, our findings identified several key challenges perceived as critical for sustaining ongoing participation. More specifically, trust in healthcare providers, accessibility of services, recurrent invitations, and informal result previews were consistently mentioned by participants. Women articulate the emotional nuances accompanying their engagement and highlight relational elements that help them recognize, regulate, and contain these emotions. This emotional containment enables them to channel feelings constructively, reinforcing their choice to maintain participation in preventive practices through a high-quality healthcare relationship. Organizational factors also play a pivotal role in encouraging women’s continued return to screening services. In particular, women emphasize the need for customization and personalization of screening experiences, underscoring that the identity of the “preventive citizen” is co-constructed through both organizational structures and relational dynamics. Beyond organizational and cognitive dimensions, the meanings uncovered in this phase imply essential emotional regulation processes. Women’s engagement is supported not only by rational assessments but also by their capacity to manage the anxiety and uncertainty inherent in preventive health experiences.

These findings align with previous research highlighting the importance of organizational and relational quality in sustaining engagement in preventive practices [21,28]. Personalized interactions, emotional containment during procedures, and continuity in communication emerged as decisive components for fostering emotional safety and promoting continued participation. The emerging categories emphasize emotional regulation, relational continuity, and organizational trust as key factors sustaining participation over time. This phase marks a critical moment in which initial motivations are tested, reinforced, or transformed through the lived experience of preventive care. Beyond internal organizational factors within healthcare services, it is important to also consider external and structural barriers that may hinder engagement—such as territorial inequalities in access to care and linguistic or cultural obstacles faced by migrant populations.

Finally, regarding the third phase of engagement, the ‘sustain’ phase—which, according to the ISE conceptual proposal, can be described as a prospective narrative focused on the future continuity of engagement—women’s narrative challenges shifted from reflecting on lived experiences to offering proactive suggestions for interventions and improvements aimed at enhancing screening services and fostering long-term participation. Continuity of Healthcare Providers, Driving Best Practices Dissemination, Flexible Organization of Healthcare Services, and Shorter Waiting Times for Results were identified as essential areas for service innovation. For women, the need to feel part of something that is considered meaningful emerges. Specifically, we believe that this coincides with perceiving the institution as interested in one’s state of health, feeling that one is respected, and feeling that one’s contribution can act as a catalyst for the involvement of other women in the care process. This phase embodies a forward-looking reconfiguration of the self in relation to prevention, where engagement is no longer episodic but integrated into a broader life narrative and healthcare trajectory. These results support the call for healthcare systems to engage citizens not only as recipients of services but as active co-constructors of preventive pathways [44,60].

By conceptualizing engagement as iterative, situated, and narrative within the cancer screening pathway, our study advances the understanding of how emotional, relational, and organizational dimensions interact dynamically over time, echoing and expanding upon previous patient engagement models [64]. The ISE perspective appears as a circular and dynamic trajectory, continuously re-signified at each step of the screening pathway. Each new experience within the preventive practice leads women to renegotiate meanings, motivations, and emotional responses. These findings suggest specific areas for clinical interventions aimed at fostering engagement, including emotional containment strategies, relational continuity, and organizational personalization to support sustainable participation. Furthermore, identifying distinct meaning-making challenges within each engagement phase provides a foundation for designing targeted interventions tailored to individuals’ phase-specific motivations and needs. For instance, women whose engagement is driven by fear-related concerns may benefit from interventions emphasizing emotional reassurance and narrative reframing, whereas those motivated by self-care goals might respond more effectively to educational and empowerment-based strategies. Recognizing these nuanced engagement profiles enables a more stratified approach to prevention, increasing the relevance, acceptability, and long-term effectiveness of screening programs.

These results align with and strengthen the theoretical perspective proposed in this study—the Iterative Situated Engagement (ISE). By demonstrating how engagement is continuously redefined through situated meaning-making processes across the recruit, retain, and sustain phases, the findings provide support for the ISE perspective. Engagement thus emerges not as a fixed state or a single decision, but as a dynamic, iterative process shaped by emotional regulation, relational experiences, and evolving interpretations of one’s role within preventive care.

The ISE framework captures the complexity of engagement as both temporally unfolding and context-dependent, emphasizing how each screening step represents a narrative turning point with specific challenges. From this vantage, targeted interventions can be crafted not only in response to behavioral profiles but also according to the shifting meanings women attribute to their engagement, thereby enhancing personalization, emotional support, and sustained participation.

## 5. Conclusions

This study enriches the field of cancer prevention research by providing a theory-driven, qualitative exploration of women’s meaning-making processes across the phases of engagement in breast and cervical cancer screening. Engagement emerges not as a static decision point, but as a circular and evolving trajectory, continuously renegotiated across screening experiences and healthcare interactions trough narrative challenges. Screening practices are not simply clinical events, but unfold as dynamic processes, deeply intertwined with women’s ongoing engagement in preventive health behaviors.

In line with the Iterative Situated Engagement (ISE) conceptual proposal, the findings suggest that fostering sustainable engagement in screening pathways requires targeted interventions across multiple interconnected levels. These levels—individual, emotional-relational, and organizational—should not be considered separate domains but rather integrated dimensions reflecting the iterative, narrative and situated nature of engagement itself. Each phase of the screening process involves a renegotiation of meanings, emotions, and narrative challenges, shaped by women’s subjectivities, their relational dynamics with healthcare professionals, and the organizational context of the services. Thus, the ISE perspective offers a flexible foundation for designing personalized clinical-psychological and organizational strategies capable of supporting the diverse engagement profiles emerging throughout the care trajectory.

This study presents some limitations. First, the sample was recruited from a single Italian region, potentially limiting the generalizability of the findings to other socio-healthcare contexts. Greater attention to socioeconomic and cultural diversity would provide a more nuanced understanding of engagement processes and barriers to accessing screening programs. Additionally, the two screening subgroups (mammography and cervical) differed in average age, which may have influenced the specific meanings and categories that emerged. Another limitation is the absence of longitudinal data; engagement was explored through one-time interviews, precluding an examination of how participation profiles evolve over time.

It is also important to highlight that cancer screening programs can carry medico-legal implications, particularly regarding informed consent and missed or delayed diagnoses. Although not central to this study, these issues deserve future attention to ensure transparency, equity, and trust in the relationship between women and healthcare services [65,66].

Future research could longitudinally investigate how the meaning-making profiles identified during the recruitment phase influence long-term engagement trajectories. Moreover, the Iterative Situated Engagement model could be applied and adapted to other preventive programs or healthcare settings to explore its validity and explanatory potential across different contexts and populations.

## Data Availability

The data presented in this study are available on request from the corresponding author due to privacy restrictions.
